# The use of matrix-specific calibrations for oxygen in analytical glow discharge spectrometry

**DOI:** 10.1007/s00216-014-8186-9

**Published:** 2014-10-22

**Authors:** Cristina Gonzalez-Gago, Petr Smid, Thomas Hofmann, Cornel Venzago, Volker Hoffmann, Wolfgang Gruner

**Affiliations:** 1 Leibniz Institute for Solid State and Materials Research (IFW) Dresden, P.O. Box 270116, 01171 Dresden, Germany; 2 AQura GmbH, Rodenbacher Chaussee 4, 63457 Hanau, Germany

**Keywords:** GD-OES, GD-MS, Glow discharge analysis, Oxygen determination

## Abstract

The performance of glow discharge optical emission spectroscopy and mass spectrometry for oxygen determination is investigated using a set of new conductive samples containing oxygen in the percent range in three different matrices (Al, Mg, and Cu) prepared by a sintering process. The sputtering rate corrected calibrations obtained at standard conditions for the 4 mm anode (700 V, 20 mA) in GD-OES are matrix independent for Mg and Al but not for Cu. The importance of a “blue shifted” line of oxygen at 130.22 nm (first reported by Köster) for quantitative analyses by GD-OES is confirmed. Matrix-specific calibrations for oxygen in GD-MS are presented. Two source concepts—fast flow (ELEMENT GD) and low gas flow (VG9000)—are evaluated obtaining higher sensitivity with the static flow source. Additional experiments using Ar-He mixtures or μs pulsed GD are carried out in ELEMENT GD aiming to improve the oxygen sensitivity.

## Introduction

The continuous progress in the development of materials motivates the need for fast, reliable, and low cost characterization techniques able to correlate the properties of materials to their composition and component distribution. High-purity materials are of particular importance for technical and scientific applications. Low contents of impurities can drastically change the properties and performance of such materials. Therefore, the determination of trace elements with high accuracy, efficiency, and low limits of detection is essential for their characterization [1]. Then, there is an urgent need to improve the procedures for the accurate characterization of high purity materials.

Solid sampling techniques such as glow discharge (GD) optical emission spectrometry (OES) and mass spectrometry (MS) are very attractive for industry and research since they allow fast and sensitive multielement analysis and do not require laborious sample treatment. The direct analysis of solid materials avoids previous dissolution and/or digestion—one of the most time-consuming steps of wet chemical analysis. Moreover, the dissolution process increases the risks of sample contamination and entails the loss of spatial information.

Similar to other solid sampling techniques, glow discharge requires calibration to accurately link the measured signal and the concentration. Due to its ability to be calibrated, carrier gas hot extraction (CGHE) is used as a standard method in the determination of oxygen in inorganic materials (metals, alloys, ceramics, etc.). Therefore, it is convenient to use this technique as reference to explore alternative methods [2]. Without calibration, GD-OES and GD-MS deliver only semi-quantitative analysis. Additionally, the use of the concepts of constant emission yields in GD-OES [3, 4] and standard relative sensitivity factors (StdRSF) in GD-MS [5, 6] are useful approximations for quantification but not applicable for metrological purposes. Consequently, the availability of SI traceable calibration material is an essential prerequisite for the reliable determination of the chemical composition of solid material by GD-OES and GD-MS. However, appropriate calibration materials are missing in many cases. The availability of SI-traceable calibration samples is particularly deficient for light elements such as H, C, N, and O. The lack of calibration material is especially noticeable for H. For C and N, a large number of steel CRMs are available with mass fractions in the range 0.0001–5 % *m*/*m* and 0.1–2 % *m*/*m* respectively. The number of CRMs is lower for oxygen and the concentration range is much smaller also in steel matrix, i.e., up to 0.01 % *m*/*m*. Additionally, some non-conductive calibration samples are available but they are not suitable for most of the commercial GD-MS instruments working in dc mode. The determination of light elements is of great importance for the characterization of primary materials in metrology since the presence of small amounts of these elements affects significantly the properties of the material. These elements dominate the impurity statement for high-purity materials, but unfortunately in most cases they cannot be accurately determined, among others, due to the lack of calibration samples. In some cases—e.g., pure Mg powder—the concentration of light elements reaches even the mass percentage range. Therefore, the production of standards at higher concentrations is essential for the accurate quantification.

The quantification of light elements is possible by analytical glow discharge spectrometry but the presence of elements such as H, C, N, or O in metals can disturb the discharge conditions producing changes on the sputtering rate, the crater shape, and the excitation and ionization mechanisms, thus having a severe influence on calibration and quantification [7–9]. If these gases are present in the samples at extremely low levels, the effects may be negligible [10].

In this context, in this preliminary work, the performance of GD-OES and GD-MS is investigated for the determination of oxygen. For this purpose, a set of new conductive samples containing oxygen concentration in the percent range in three different matrices (Al, Mg, and Cu) was produced by a sintering process. Variations on sputtering rates and discharge parameters with oxygen concentration are evaluated. Furthermore, matrix-specific calibrations of oxygen are reported. On the basis of such investigations, it may be possible in the future to perform more accurate semi-quantitative or even quantitative oxygen analysis. In GD-OES, it is not yet known if matrix-independent calibration is possible for the light elements [11]. Because of the low sensitivity of all commercial GD-OES instruments for oxygen, in most cases, the calibration has been based on the sample CE650 from Jernkontoret (32.4 % *m*/*m* oxygen) which was the only available high-point calibration sample for oxygen. However, this sample is now out of stock and there is no other calibration material with high oxygen concentration available. Sometimes, uncertified home-made samples, as, e.g., a layered hot rolled steel sample with some content of Fe_3_O_4_ (25 % *m*/*m* oxygen), are used for calibration. A typical calibration of oxygen is shown in [12]. Scattering and emission yield depend on the selected calibration samples and we will show in this paper that the emission yield of oxygen at 130.22 nm depends on the sample matrix, if spectrometers with a spectral resolution of about 20 pm or worse are used. In principle, samples with lower oxygen content can be also analyzed by GD-OES, if special high vacuum sources and spectrometers are used. A background equivalent concentration (BEC) of 100 μg/g with only 3–4 % RSD can be achieved [13]. GD-MS measurements were carried out on two commercially available double-focusing spectrometers. One coupled to a static flow cryo-cooled (VG9000) and another one using a fast flow source with Peltier cooling (ELEMENT GD). In GD-MS, oxygen has the lowest sensitivity compared to other elements and a strong dependence of this sensitivity on the matrix and plasma conditions is known (RSF_Al_ = 6 and RSF_Zr_ = 50) [14]. No limits of detection (LOD) are reported in this paper, but oxygen concentrations below 1 μg/g are given. Overnight pumping and a long presputter time were applied to reduce the background. As far as the oxygen, carbon, and nitrogen background level on ELEMENT GD is concerned, it was reported that it can be reduced by applying heating and/or cooling of the sample and by purging the GD source during the sample exchange [15]. This approach was not applied in this study.

This research work contributes to the European Metrology Research Program (EMRP)—project SIB 09 which pursues the production of Primary Standards for Challenging Elements [16].

## Experimental

### Sample selection and production

For our experiments Cu, Al, and Mg were selected as matrices. Cu is a well-known host matrix for the analysis of powder samples in glow discharge [17]. Al and Mg are of great importance for the industry for light weight construction materials as used in automotive and aerospace industry.

A set of samples with a defined content of oxygen was prepared by sintering of powder mixtures. The sintering technology has shown to be adequate for the production of electrically conductive calibration materials, but the right raw material and technological parameters still must be found, especially the pressure/temperature-time profile. In order to use this material for calibration in GD spectrometry, the concentrations of all elements in the samples must be known. When the standard Grimm-type source is used, the samples also should be vacuum tight. For the application of the so-called multi-matrix calibration, well proven in GD-OES, the sputtering rate must be known/determined.

Sintering of the Mg and Al compacted powders was carried out under 0.5 atm Ar at 400 °C and a pressure of 150 kN. The sintering temperature of Cu was 600 °C. The temperature of the samples was increased and decreased with a speed of 20 K/min and kept constant at sintering for 20 min. The used press Type PWV30EDS was built by Paul-Otto Weber Maschinen-Apparatebau GmbH, Remshalden, Germany.

To prepare the set of Cu samples, different amounts of CuO (0.5, 1, and 2 g) were mixed with 10 g of Cu powder. The same amounts (0.5, 1, and 2 g) of Al_2_O_3_ and MgO were added to Al and Mg powder, respectively. Because of the low density of these matrices, the oxides were added to only 6 g of the matrix. Additionally, we added about 5 % *m*/*m* Mg to the Al samples and about 5 % *m*/*m* Al to the Mg samples in order to compare the ratio of the oxygen signal to the Mg and Al signal versus the concentration ratio for both matrices. A list of the samples used for the calibration is included in Table 1.

**Table 1:** List of samples used for the calibrations

Cu-matrix	Al-matrix	Mg-matrix
Bulk Cu	Bulk Al	Bulk Mg
10 g Cu	6 g Al	6 g Mg
10 g Cu + 0.5 g CuO	6 g Al + 0.3 g Mg + 0.5 g Al_2_O_3_	6 g Mg + 0.3 g Al + 0.5 g MgO
10 g Cu + 1 g CuO	6 g Al + 0.3 g Mg + 1 g Al_2_O_3_	6 g Mg + 0.3 g Al + 1 g MgO
10 g Cu + 2 g CuO	6 g Al + 0.3 g Mg + 2 g Al_2_O_3_	6 g Mg + 0.3 g Al + 2 g MgO

In order to determine the instrumental background for oxygen, bulk material was included in the calibration. In the GD-OES experiments we used electrolytic Cu (99.9 % *m*/*m*), Mg (99.8 % *m*/*m*) from Alfa Aesar (Ward Hill, MA, USA, Product Nr. 10231) and for Al HA-5 (99.9 % *m*/*m*) from Research, Engineering and Prime Contracting Centre of the Hungarian Aluminium Corporation. Even if the concentration of oxygen in these bulk samples is not known, it is far below the LOD of the GD-OES instrument. In the GD-MS experiments, the samples employed to establish the oxygen background were Al (99.99 % *m*/*m*) from Hydro (Germany, Product 22891), electrolytic Cu (99.99 % *m*/*m*) from Community Bureau of Reference—BCR (Product CRM-074A) and Mg (99.8 % *m*/*m*) from Alfa Aesar (Ward Hill, MA, USA, Product Nr. 10231).

### Instrumentation and characterization techniques

GD-OES measurements were carried out at IFW Dresden with the commercial spectrometer GDA750 (Spectruma Analytik GmbH, Hof, Germany), which measures the intensity of the spectral lines by photomultipliers. A standard Grimm-type 4-mm dc source was used in continuous dc mode for all experiments. Line profiles were measured by scanning of the entrance slit. Argon (99.999 % minimum purity) from Praxair was used for GD-OES analysis.

For GD-MS experiments, two commercially available instruments at AQura GmbH were used: the VG9000 (VG Elemental Ltd., Winsford, GB, UK) and ELEMENT GD (Thermo Fisher Scientific, Bremen, Germany) both based on double focusing mass spectrometers. A cryo-cooled plasma cell for flat samples was used on the VG9000 instrument. This source is a modified version of the initial source in VG9000 with enlarged sputtering area (∅ 10 mm) [18]. The GD source on the ELEMENT GD is a Grimm-type chamber consisting of an anode with 8 mm diameter and an internal flow tube cooled by Peltier elements. The latter instrument can be operated in continuous as well as in μs-pulsed (PGD) dc mode. For the operation in PGD, an external high voltage power supply (RUP-3A, GBS-Elektronik GmbH) was connected to the discharge chamber via the same channel as the usual dc power supply. The experimental set up employed for pulse generation has been described elsewhere [19]. Additionally, this instrument offers the possibility of adding alternative gases. VG9000 can be only operated in continuous dc mode. Argon (99.999 % minimum purity) from Messer Griesheim GmbH (Germany) and helium (99.999 % minimum purity) from Linde (Germany) were employed as discharge gases for ELEMENT GD and argon (99.99999 % minimum purity) from Linde for VG9000. An additional gas purifier (MEGASORB, Messer Griesheim GmbH) is used in this instrument.

The sputtering rate (SR) measurements were undertaken during the GD-OES experiments. The crater volume was measured with the profilometer MicroProf (FRT, Bergisch Gladbach, Germany). This profilometer measures the topography of the crater based on the principle of chromatic aberration providing an accuracy in the lower percent range, but the reproducibility depends on the preparation of the sample surface. More severe deviations are known at sample exchange and most probably also caused by not ideal sample surface and thus slightly different positions of the samples on the source. The relative error of the sputtering rates is estimated to be about 10 % [20].

The determination of the total oxygen concentration in the calibration samples for GD investigations was carried out by CGHE in helium atmosphere. A TC436-DR analyzer (Leco, USA) was used in the double-IR-detector mode to measure both reaction gases CO and CO_2_. These gases may be formed in the basic carbothermal reduction reaction of oxygen species with the carbon crucible material from the sample during CGHE process. Depending on the possible reaction mechanism, this option must be applied in the case of the precise oxygen determination in many oxides or, in general, in the case of large amounts of oxygen in analyzing samples [21]. Besides the common impulse heating technique in CGHE for small or low oxygen concentrations, the release of large volume of gaseous reaction product CO_*x*_ in that case requires a softer heating process realized as ramping of 2000–4000 W with 100 W/s in the presented case.

The determination of oxygen by CHGE was limited to copper of the type CuO/Cu-matrix since for Al- and Mg-matrix significant inaccuracies are expected. As usual, measurement conditions at high temperatures necessary for the carbothermal reaction applied to Al_2_O_3_ and MgO evaporation of the metallic Al- and Mg-matrix occurs followed by possible gettering effects during deposition at colder surfaces in the apparatus. Therefore, an analyte yield <100 % and thus inaccurate results have to be expected. From the sintered samples, pieces have been mechanically cut. The initial weight of samples with 0.5–2 g of oxide was 100–500 mg, but for oxide-free sintered Cu samples, about 1 g was used. All sintered samples were measured directly, i.e., without further melting additions. The independent calibrations of the IR detectors were realized using gas dosing with pure CO_2_ gas, pure ZrO_2_ and solid state reference material ZRM-ON5.

## Results and discussion

### Characterization of sintered samples

In this section, results of the characterization of the sintered samples used for the ongoing investigations are presented. At this early stage of the study, the extensive validation by reference analytical techniques is still in progress.

Table 2 collects the oxygen concentration measured by CGHE analysis for the set Cu samples employed in this work. Theoretical values calculated from the amounts of raw materials added for sample preparation are also given. For pure Cu, it is observed that the pressed samples containing CuO have a content of oxygen quite higher than expected from the added CuO. Whereas the sintered sample of pure Cu powder contains only 150 μg/g oxygen, the pure Cu powder and the sintered mixtures with CuO show an about 1 order of magnitude higher difference. The quality of the prepared sintered samples depends on two important factors: the use of powder as starting material, and the application of heat during sintering process. The untreated Cu powder has a larger surface than a sintered sample and the surface may be covered by some oxygen-containing species as by humidity and oxidation products [22], depending on storage conditions. In the heating process during sintering, the amounts of surface-bound oxygen species can be decreased from 0.186 to 0.015 % *m*/*m* oxygen as demonstrated. This effect becomes more significant at low than at high oxygen concentrations in the oxide containing samples. The CGHE method can serve as a reference method for oxygen determination of the given copper samples, but also as testing method in the preparation process of samples.

**Table 2:** Oxygen concentrations in sintered Cu samples: theoretical (calculated) and measured (by CGHE) values are given together with the ratio of measured and theoretical density

Sample	O concentration calculated (% *m*/*m*)	O concentration measured (CGHE) (% *m*/*m*)	Error (% *m*/*m*)	Difference	Density measured/theoretical (%)
Cu powder	0	0.186	0.009	0.186	
10 g Cu (sintered)	0	0.015	0.001	0.015	97.5
10 g Cu + 0.5 g CuO	0.958	1.071	0.003	0.113	93.2
10 g Cu + 1 g CuO	1.829	1.944	0.011	0.115	93.1
10 g Cu + 2 g CuO	3.352	3.578	0.011	0.226	88.6

These data show clearly that it is impossible to predict the amount of oxygen, introduced by the pure metallic powders itself. Because of the production under inert atmosphere, it is most probably that the upper limit of this contribution is near the concentration of oxygen in the corresponding metal powder. However, this value is not a real constant and may increase with time or, e.g., handling in ambient atmosphere. A reduction of this concentration by the sintering procedure is possible (see sintered Cu powder), but not inevitable.

**Fig. 1:**
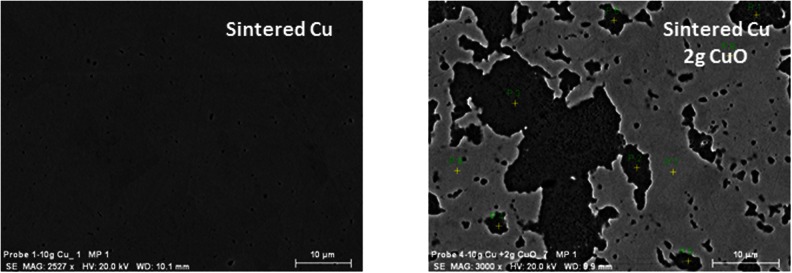
SEM images of pure Cu (*left side*) and Cu + CuO (*right side*) sintered samples

The images in Fig. 1 show the surface of two sintered Cu samples; sintered pure Cu (left side) and the sample consisting of 10 g of Cu powder and 2 g of CuO. As can be seen, the pure sample has a homogeneous surface. However, in the sample with CuO, two different regions are observed corresponding to Cu and Cu oxide respectively. Additionally, in the dark area associated with Cu oxide, certain porosity is observed. This explains the fact that the density, determined from the mass and volume of the samples, results in 88–97 % of the theoretical value, calculated from the amounts of powder weighed for sample preparation.

The determination of oxygen in Al- and Mg-matrix sample is still in progress and the weighed oxygen concentrations were used in the data evaluation up to now.

As mentioned above, the analysis of oxygen in Mg-based or Mg-containing samples by CGHE is a particularly complex task. Therefore, another set of Al samples prepared without any Mg content will be used later in order to compare the sensitivity between Al and Cu more accurately.

### Oxygen determination by GD-OES

All prepared sintered samples were measured at standard conditions for the 4-mm dc source (700 V, 20 mA, Ar) together with the cast pure bulk sample of Cu, Al, and Mg. In order to determine the background on both sides of the weak oxygen line O I 130.22 nm, the emission spectrum was scanned in a narrow wavelength range by moving the entrance slit. In the same scan, the intensities of the lines N I 149.26 nm, C I 156.14 nm, Al II 167.08 nm, Mg I 383.83 nm, Al I 396.15 nm, Cu II 219.23 nm, Cu I 327.40 nm, and Ar I 415.86 nm were also measured.

**Fig. 2:**
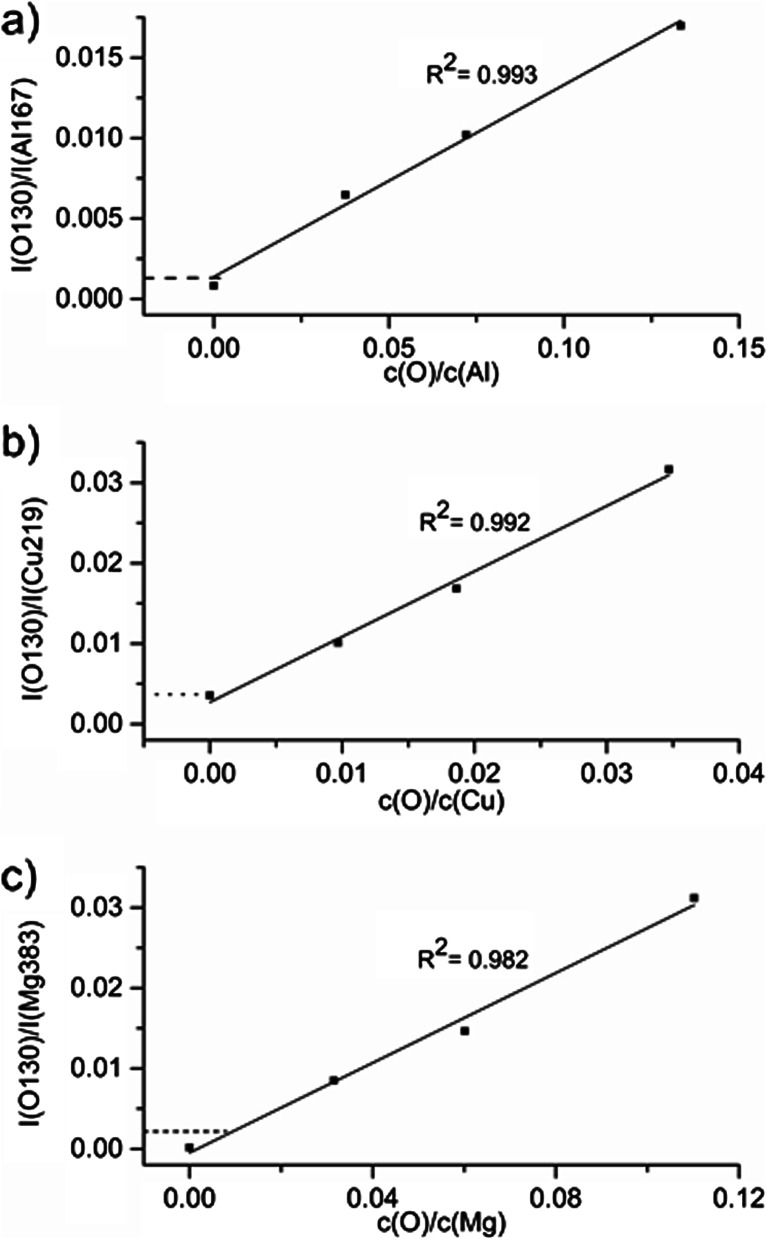
Matrix-specific calibration for oxygen in GD-OES: **a** Al-matrix; **b** Cu-matrix; **c** Mg-matrix. The samples were measured at standard conditions for the 4-mm dc source (700 V, 20 mA). The *horizontal lines* represent the relative intensities corresponding to the pure sintered samples. Note that different scales are used

The matrix-specific calibrations are presented in Fig. 2. In this form of calibration, the ratio of the intensities of oxygen and matrix lines is calculated after background subtraction and plotted versus the ratio of concentrations. In this way, changes in sputtering rates are corrected if the intensities depend linearly on the sputtering rate. The background of the oxygen line was measured at the higher wavelength part of the line profile (this will be discussed at the end of this section). For all samples, the initially weighed concentrations were used, which means that only the intentionally added oxygen from the oxides is taken into account. No error bars are shown in Fig. 2. However, the estimated relative error based on the multiple measurements on one crater is <10 %. The measurement at different positions of the sample however can cause a higher RSD because of sample inhomogeneity and small leakages. Additionally, the background concentrations of light elements improve during operation and degrade again afterwards. This could be the cause for higher oxygen background observed on the Cu samples where theoretically no oxygen should be present in comparison to the Mg and Al samples, measured also in this sequence. Investigations to reduce and quantify these additional sources of error are ongoing and will be published in due course. The relative intensities corresponding to the pure sintered samples were not considered for the calculation of the linear fitting since these samples contain a certain amount of oxygen (not voluntarily added). Nevertheless, these values are marked by horizontal lines in Fig. 2. Even if the exact concentrations of oxygen in the sintered pure Al and Mg samples is still not known, it is observed that they are at a higher level than in the sintered pure Cu sample (~150 μg/g determined by CGHE).

All curves are linear and matrix-independent calibration was therefore investigated. For this purpose, the sputtering rates of all samples were measured for 20 mA, 500, 700, and 900 V. According to Boumans [23], it was observed that the reduced sputtering rate show a linear dependence on voltage.

The sputtering rates decrease with the increase of the oxygen concentration due to the presence of oxide particles in the material, which are sputtered slower in GD. The sintered Al sample without addition of an oxide has the highest sputtering rate, whereas the Mg and Cu bulk samples show higher sputtering rates than the sintered samples. The curves also were used to confirm the estimation of the error of the sputtering rate measurement.

**Fig. 3:**
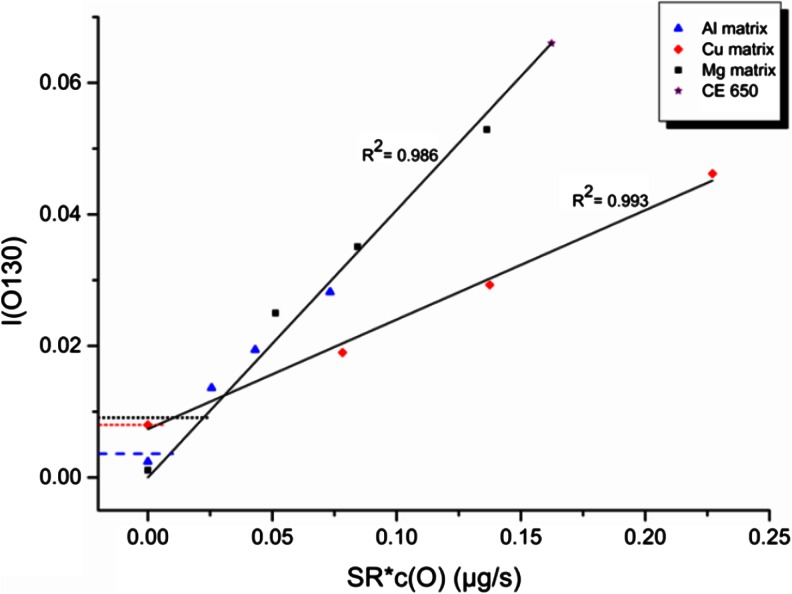
Oxygen calibration (corrected by sputtering rates) in GD-OES. The samples were measured at standard conditions for the 4-mm dc source (700 V, 20 mA). The *horizontal lines* represent the intensities corresponding to the pure sintered samples (*short dashed line* for Al, *dashed line* for Cu, and *dotted line* for Mg)

The resulting sputtering rate corrected calibration is shown in Fig. 3. Besides the data of the three matrices Cu, Al, and Mg this figure also includes the point of the well know highpoint calibration sample CE 650 from Jernkontoret (32.4 % *m*/*m* oxygen). This calibration material with higher concentration of oxygen is used to overcome the problems of the exact determination of the light element at low levels. The horizontal lines represent again the intensities of the pure sintered samples. Equally in Fig. 3, no error bars are shown. The relative error estimated based on several measurements is <10 %.

Firstly, the scattering of the points corresponding to samples showing no significant concentration of oxygen should be mentioned and discussed. As it was already mentioned for the matrix-specific calibration of oxygen, the presence of small leakages can enhance the intensity of oxygen contributing to the scattering of the low points but also for higher concentrations. Secondly, a real oxygen concentration around 0.5 % *m*/*m* is expected in the pure sintered Mg and Al samples.

The second and main result of this evaluation consists of the fact that except for Cu all samples follow a matrix-independent calibration curve. It is known that this is also the case for iron oxide, but this material is not available as standard and mostly used as a layered material.

The deviation of the Cu curve is very obvious and must be discussed in detail, because this means that matrix-independent quantification will fail in some cases.

**Fig. 4:**
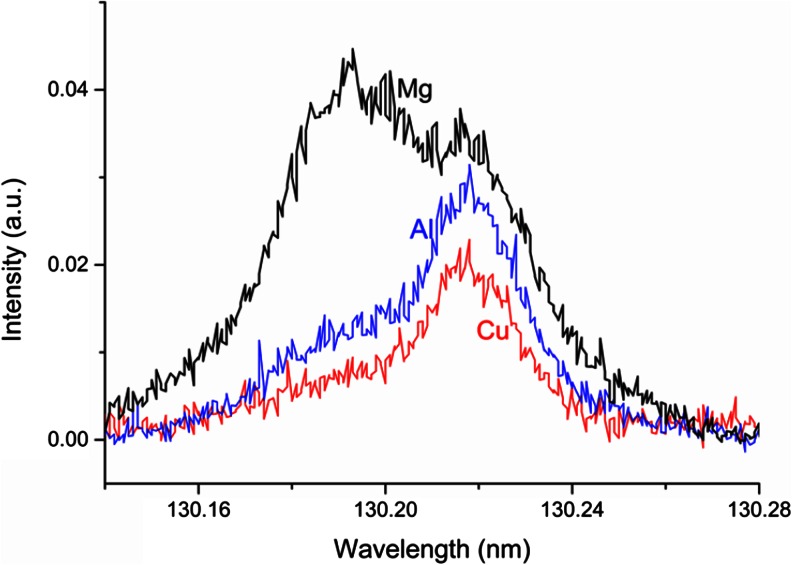
Oxygen 130.22 nm line profile for three samples with sputtering rate of about 0.08 μg/s

**Fig. 5:**
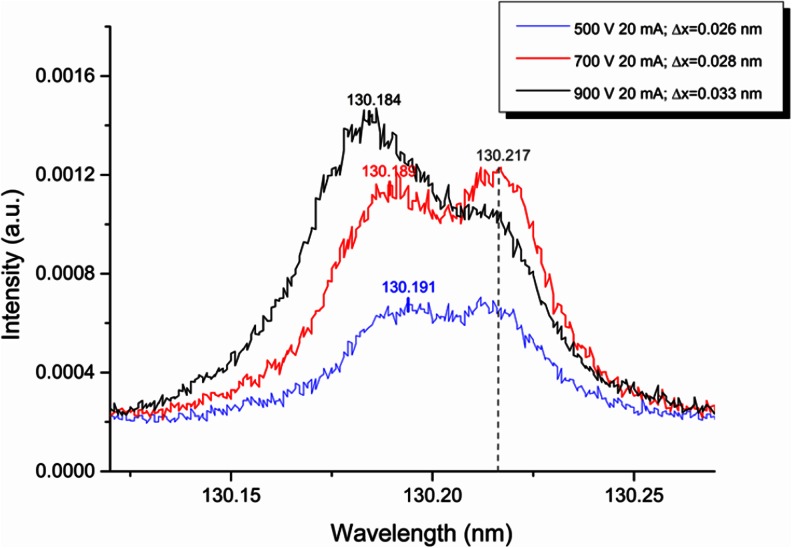
Oxygen 130.22 nm line profile at different voltages (500, 700, and 900 V) for the sample with 6 g Mg and 2 g MgO sputtered in Ar

Figure 4 shows the oxygen 130.22 nm line profile of the three samples containing oxygen with sputtering rate of about 0.08 μg/s (red curve for 10 g Cu + 0.5 g CuO, blue for 6 g Mg + 1 g MgO and black for 6 g Al + 2 g Al_2_O_3_). Clearly, there is a second line at lower wavelength, which interferes with the O I 130.22 nm line. This contribution is smallest for the Cu sample. After interference correction, the emission yield (defined as the ratio of intensity and the product of the concentration and the sputtering rate) of the different matrices are very close together. However, the accurate correction of such interferences is difficult due to the high noise of the signals. Considering the integrated area of the complete wavelength range at both emission lines did not show any improvement in terms of matrix independent calibration. One possible explanation for this additional emission line is the blue shift of the O I 130.22 nm line. This effect in GD-OES has been reported by Michael Köster [24]. Unfortunately, the origin of this effect is not fully understood and nothing was published about it yet. Nevertheless, our present investigations confirm this effect and demonstrate the importance for quantitative analyses by GD-OES. We found that the line shifts to the blue side proportionately to the square root of the applied voltage (see Fig. 5), which points to the Doppler Effect as suggested by Michael Köster. However, we could not find this effect with a similar size at the three lines at 777 nm. Furthermore, a low pressure causes a higher ratio of the shifted to the non-shifted line, which can be related to an extended cathode dark space. Therefore, the effect becomes more and more essential at materials with high secondary electron emission yield as Al and Mg, where the plasma show the same voltage and current but at a lower pressure. The Ar pressure in the GD-OES experiments decreased from about 12.5 hPa at Cu over about 10.7 hPa at Al and finally to 8.7 hPa at Mg. Higher oxygen concentration also enhances the secondary electron emission of the Cu and Mg samples. Therefore, the pressure of the experiments with the samples with highest oxygen concentration was about 0.5 hPa lower than when measuring the bulk sample. For Al, the pressure was nearly independent of the oxygen concentration.

Because of the blue shift of the 130.22 nm oxygen line and its low intensity, it is essential to determine the background at the higher wavelength side of the line and to correct for this background.

### Oxygen determination by GD-MS

The analysis of light elements in GD-MS is known for the static flow source at the VG9000 but there is little or no knowledge concerning detection limits, sensitivity, and reproducibility of light element determination with instruments using fast flow sources. Therefore, most of the experiments here reported are focused in the evaluation and optimization of the fast flow source concept in the ELEMENT GD for oxygen determination.

#### Evaluation of plasma parameters for the fast flow GD source

In dc glow discharge, the plasma conditions are defined by three parameters flow, current, and voltage. In case of ELEMENT GD, flow and current are typically fixed. Thus, different current and flow conditions were evaluated aiming to get the maximum oxygen to matrix intensity ratio. For these experiments, two samples were selected as representative of each matrix (a reference material without oxygen and the sintered sample with the highest oxygen concentration). Variations of sensitivity are represented by the slope obtained by plotting the ion beam ratios corrected for the relative abundances of ions (IBR) versus oxygen to matrix concentrations. The slopes and voltages obtained under the plasma conditions investigated are given in Table 3. Note that the reciprocal value of the slope of such a plot is called relative sensitivity factor (RSF) in GD-MS.

**Table 3:** Slopes and *y*-intercepts obtained by representing IBR versus oxygen/matrix concentration under: different current conditions at 320 sccm and different gas flows at 50 mA. The voltages corresponding with the investigated current-flow conditions are also given

Different current conditions at 320 sccm						
Current (mA)	50	60	70	80		
Al-matrix	Slope	11088	11535	11792	12080	
	*y*-intercept	31.51	68.10	44.17	40.02	
	Voltage (V)	751	801	856	940	
Cu-matrix	Slope	11151	13964	13707	–	
	*y*-intercept	12.03	10.73	11.24	–	
	Voltage (V)	1061	1157	1192	–	
Mg-matrix	Slope	20810	22678	24027	26413	
	*y*-intercept	39.09	29.53	22.45	17.60	
	Voltage (V)	427	497	573	704	
Different gas flows at 50 mA						
Flow (sccm)	320	340	360	380	400	
Al-matrix	Slope	11088	11016	10454	12096	11944
	*y*-intercept	31.51	36.52	35.35	40.02	79.20
	Voltage (V)	751	773	718	668	616
Cu-matrix	Slope	11151	12470	13020	14853	17095
	*y*-intercept	12.03	14.39	17.46	12.84	16.21
	Voltage (V)	1061	1005	974	896	846
Mg-matrix	Slope	20810	20409	15334	15444	14024
	*y*-intercept	39.09	236.10	219.26	247.68	308.03
	Voltage (V)	427	413	399	369	346

Firstly, the effect of current was evaluated fixing the flow at 320 sccm while the current ranged between 50 and 80 mA. As shown in Table 3, the sensitivity (represented by the slope) and the voltage increase with current for all the investigated matrices. However, in the case of Cu, noticeable instabilities were observed at 70 mA and at higher currents shortcut effects occur due to the high sputtering rate and re-deposition.

Secondly, the current was fixed at 50 mA while the flow was adjusted from 320 to 400 sccm. For all matrices, it is observed that the voltage decreases when the flow is increased (see Table 3). However, in terms of oxygen sensitivity, the effect of flow is different for each matrix. For Cu samples, the sensitivity of oxygen increases with the gas flow while it decreases for Mg; no clear tendency is observed in the case of Al.

#### Matrix-specific calibration

The set of samples with a defined concentration of oxygen collected for the calibration were measured in the ELEMENT GD in continuous mode, using Ar as plasma gas under the conditions typically used for the respective matrices (320 sccm, 80 mA for Al, 400 sccm, 60 mA for Cu and 320 sccm, 80 mA for Mg).

**Fig. 6:**
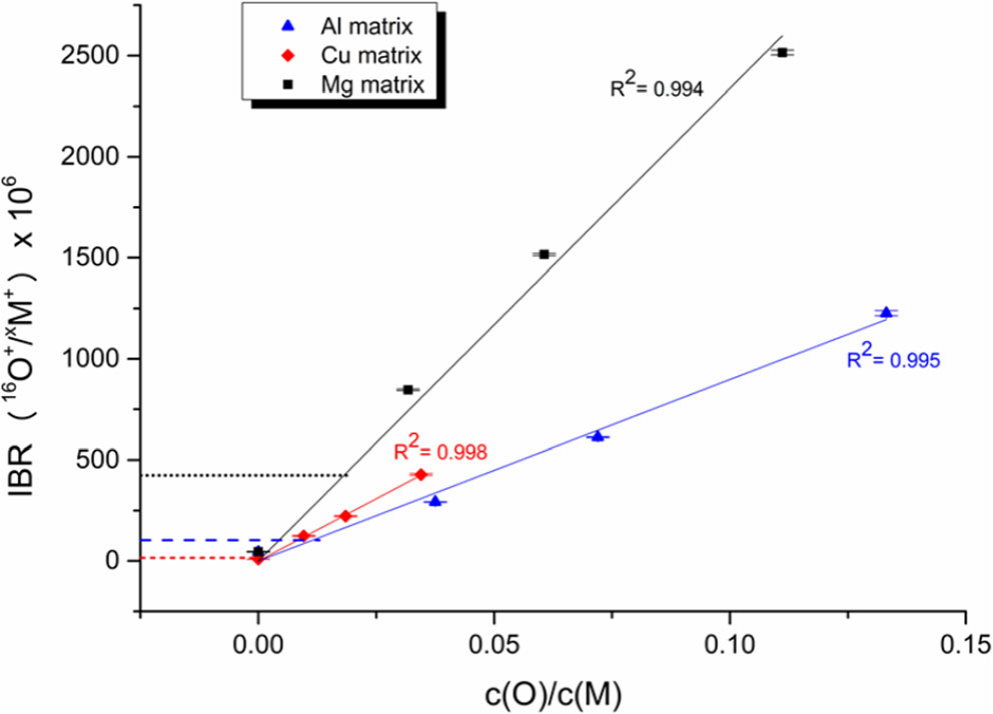
Matrix-specific calibration for oxygen in GD-MS for Al-matrix (320 sccm, 80 mA), Cu-matrix (400 sccm, 60 mA), and Mg-matrix (320 sccm, 80 mA). The *horizontal lines* represent IBR obtained in the pure sintered samples (*short dashed line* for Al, *dashed line* for Cu, and *dotted line* for Mg)

Figure 6 plots the specific calibration obtained for oxygen in the three investigated matrices. As in GD-OES experiments, the pure sintered samples were not included in the calibration but the measured values are indicated by the horizontal lines. As can be observed in the figure, good linearity is obtained in all cases. In terms of oxygen sensitivity, matrix dependence is observed: the oxygen sensitivity is two times higher in Mg than in Cu and Al.

#### Comparison of ELEMENT GD and VG9000 for oxygen determination

As a comparison, the performance of the static flow source concept in the VG9000 has been evaluated for oxygen determination in GD-MS. For these experiments, the set of sintered Cu samples with a defined content of oxygen were measured under conditions selected as optimum for each of the instruments (60 mA, 400 sccm for ELEMENT GD and 5 mA, 1 kV for VG9000). A benefit of the cryo-cooling on the VG9000 is the reduction of gas background (for cast pure Cu, the IBR of oxygen is around eight times lower on the VG9000 than on the ELEMENT GD) as well as improved stability of the source [6] at constant temperature. On the other hand, the fast flow source of the ELEMENT GD permits higher currents (up to 90 mA in contrast to 10 mA for VG9000) and higher argon gas flows leading to more effective sample sputtering [25].

ELEMENT GD is operated at constant flow and voltage settings because it has been observed that this mode provides better analytical performance. In VG9000, similar performance is obtained when it operated on fixing flow and voltage or voltage and current. Nevertheless, due to safety reasons, VG9000 is generally operated under fixed voltage and current. In this respect, current is fixed while flow is manually changed to achieve the desired voltage. On the other hand, different gas purities are used on both instruments motivated by the different gas consumptions. The 7 N Ar typically used at VG9000 cannot be used in ELEMENT GD due to the high cost.

**Fig. 7:**
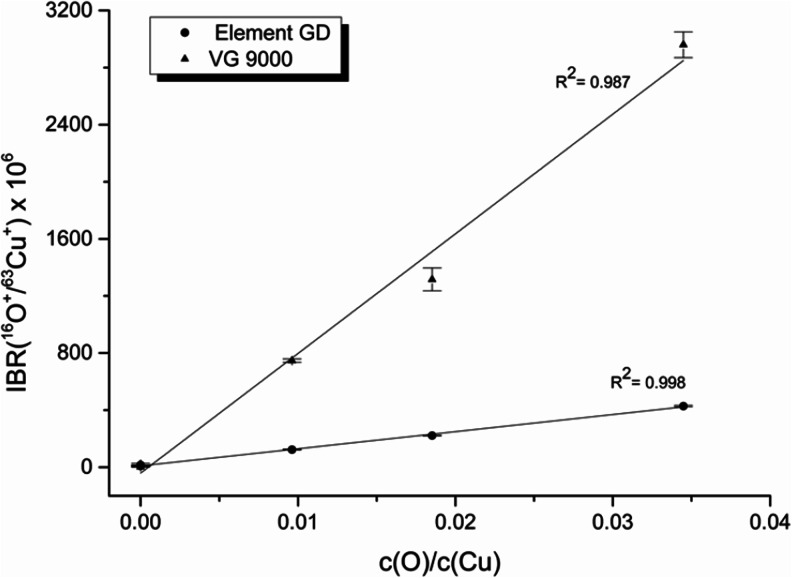
Comparison of oxygen sensitivity in VG9000 and ELEMENT GD. The sintered Cu samples were measured in both instruments under optimum conditions (400 sccm, 50 mA for ELEMENT GD and 5 mA, 1 kV for VG9000)

The respective sputtering rates were measured finding values 1 order of magnitude lower in the case of the VG9000. Nevertheless, the sensitivity obtained with such an instrument is more than six times higher than that in the ELEMENT GD (see Fig. 7). However, even the higher sensitivity of oxygen at the VG9000 is still quite low compared to metallic elements and therefore, the determination of matrix-specific sensitivity factors of oxygen is essential.

Aiming to improve the sensitivity for oxygen, additional possibilities of ELEMENT GD such as PGD or Ar-He mixtures were evaluated.

#### Evaluation of the pulsed dc mode

By contrast with the continuous mode, the pulsed GD is operated at constant flow and voltage. Flow and voltage as well as pulse parameters were optimized to get the best performance in terms of oxygen sensitivity. However, it has been observed that the oxygen sensitivity is lower in the pulsed mode in all cases because the resulting mean power is lower than in the continuous mode.

It is known that the use of pulsed GD offers certain advantages such as reduced thermal effects, enhanced atomization, excitation, and ionization by application of high, short-term power [26]. However, in the ELEMENT GD, the current in the pulse mode is not high enough. In previous investigations carried out by Voronov et al. [19], it was observed that electrical currents in microsecond PGD depend on the cell geometry. In the ELEMENT GD, the discharge chamber consists of a wide tube (Ø 5 mm) connecting the discharge area and the skimmer, and all the gas is flowing through it. Thus, the gas pressure is lower than in other discharge chambers resulting in lower electrical current.

#### Effect of He addition on oxygen sensitivity

One of the reasons why the determination of light elements by GD-MS is difficult is related to their high ionization potential (13.61 eV for oxygen). Argon is the gas commonly used to generate a glow discharge due to its relative low cost. The metastable energy levels of 11.55 and 11.72 eV are high enough to ionize most of the elements in the periodic table. However, several non-metallic elements such as hydrogen, nitrogen, and oxygen have higher ionization potential. Therefore, these elements cannot be ionized by Penning mechanisms in an Ar discharge. The use of alternative gases such as helium with more energetic metastable levels (19.82 eV and 20.62 eV) could improve the ionization but the sputtering efficiency is noticeably reduced due to its lighter mass. Therefore, Ar-He mixtures are potentially more interesting combining the sputtering capabilities of Ar with the high energy levels of the He metastables [27]. Studies carried out by Lange et al. [28] had shown that sensitivities for non-metals are enhanced when He is added to the plasma.

The effect of He addition to the Ar plasma gas was evaluated in terms of oxygen sensitivity. These investigations were carried out using the set of sintered samples with defined concentration of oxygen in Al-matrix.

**Fig. 8:**
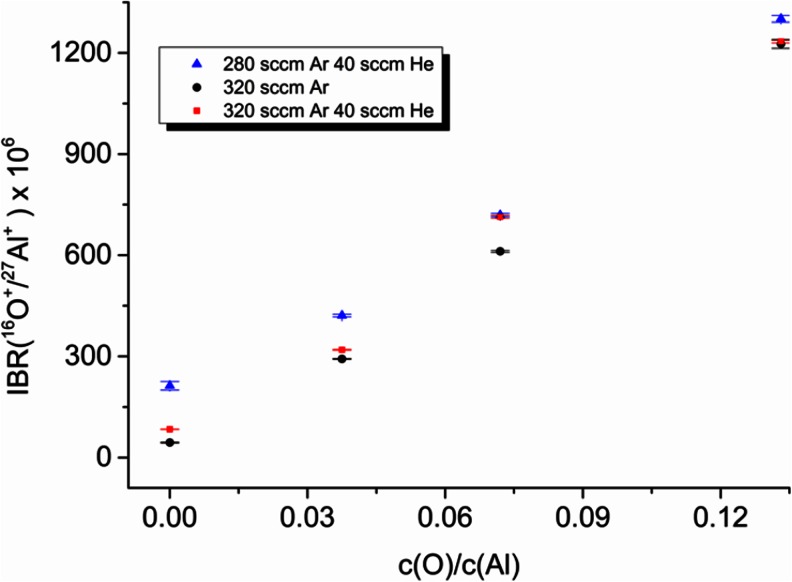
Effect of He addition on oxygen sensitivity

In the first stage, the Ar and He flows were fixed at 280 and 40 sccm, respectively, obtaining a total gas flow of 320 sccm, which is the flow used for routine analysis of Al samples in pure Ar. In such a situation, the voltage increases considerably and it is not possible to evaluate higher concentrations of He because the maximum voltage is reached and then the fixed current (80 mA) cannot be achieved. Afterwards, aiming to reduce the voltage, the Ar flow was maintained at 320 sccm while the He concentration was increased to 40 sccm resulting in a total flow of 360 sccm. The oxygen calibration curves obtained in pure Ar and the above mentioned gas mixtures are shown in Fig. 8. As can be seen, the variations of sensitivity are not significant (<4 %). It should be mentioned that Lange [28] observed improvements of sensitivity for He concentrations between 20 and 30 % *v*/*v*. However, in our experiments, we cannot go above 15 % *v*/*v* of He without modifications of the current and flow conditions.

## Conclusions

It is demonstrated that the samples prepared by sintering of powder mixtures are adequate for the analysis by GD-OES and GD-MS. In addition, the prepared samples provide linear matrix-specific calibrations for oxygen.

For GD-OES, under the same voltage and current conditions, the O I 130.22 nm line has a lower emission yield in Cu than in Al and Mg. For Al and Mg matrices, the intensity of the O I 130.22 nm line is affected by a blue-shifted line which increases its emission yield. For these two matrices, the gas discharge pressure required to maintain the voltage and the current constant is lower than for Cu due to the high secondary electron emission of Al and Mg. At lower gas pressures, the blue shift effect seems to be more pronounced. It has been observed that the oxygen lines at 777 nm do not suffer from the spectral interferences due to this blue shift phenomenon and will therefore be used in further studies for more accurate results.

In GD-MS, relatively low oxygen sensitivity is observed compared to other elements. Pronounced differences are also observed between both GD-MS plasma cell concepts; the fast flow-based GD concept of ELEMENT GD shows approximately six-times-lower sensitivity than the static flow GD concept of VG9000. This poor sensitivity on ELEMENT GD is not improved by the addition of alternative discharge gases with more energetic metastable levels such as He at concentrations lower than 15 % *v*/*v*.

Furthermore, in the current experiments, an apparent matrix-specific oxygen calibration is shown in GD-MS, i.e., the IBRs, calculated as the ratio between oxygen and matrix intensities, and thus the slope of the calibration curve depends on the sensitivity not only of oxygen but also of the matrix element (lower for Mg than for Al and Cu). Preliminary results comparing the ratio of the oxygen signal to the Mg and Al signal versus the concentration ratio for both matrices recorded with the sintered Al samples containing approx. 5 % *m*/*m* of Mg and with the Mg-sintered samples containing approx. 5 % *m*/*m* of Al indicate that matrix-independent oxygen calibration may be possible. However, accurate determination of matrix-specific sensitivity factors for oxygen is essential for establishment of a multi-matrix calibration. Further studies including sputtering rate corrections are needed to evaluate the possibility of matrix independent calibration for oxygen in GD-MS. Therefore, the best set of discharge conditions must be found and the effect of the variation of the third discharge parameter on the sensitivity of oxygen must be understood. Sputtering rate measurements should be included to separate the changing intensities of oxygen and matrix. Further detailed results of the ongoing investigations will be published in due course.
